# Conditional Knockout of Prolyl Hydroxylase Domain Protein 2 Attenuates High Fat-Diet-Induced Cardiac Dysfunction in Mice

**DOI:** 10.1371/journal.pone.0115974

**Published:** 2014-12-29

**Authors:** Heng Zeng, Jian-Xiong Chen

**Affiliations:** Department of Pharmacology and Toxicology, University of Mississippi Medical Center, Jackson, Mississippi, 39216, United States of America; University of Cincinnati, College of Medicine, United States of America

## Abstract

Oxygen sensor prolyl hydroxylases (PHDs) play important roles in the regulation of HIF-α and cell metabolisms. This study was designed to investigate the direct role of PHD2 in high fat-diet (HFD)-induced cardiac dysfunction. In HFD fed mice, PHD2 expression was increased without significant changes in PHD1 and PHD3 levels in the heart. This was accompanied by a significant upregulation of myeloid differentiation factor 88 (MYD88) and NF-κB. To explore the role of PHD2 in HFD-induced cardiac dysfunction, PHD2 conditional knockout mice were fed a HFD for 16 weeks. Intriguingly, knockout of PHD2 significantly reduced MYD88 and NF-κb expression in HFD mouse hearts. Moreover, knockout of PHD2 inhibited TNFα and ICAM-1 expression, and reduced cell apoptosis and macrophage infiltration in HFD mice. This was accompanied by a significant improvement of cardiac function. Most importantly, conditional knockout of PHD2 at late stage in HFD mice significantly improved glucose tolerance and reversed cardiac dysfunction. Our studies demonstrate that PHD2 activity is a critical contributor to the HFD-induced cardiac dysfunction. Inhibition of PHD2 attenuates HFD-induced cardiac dysfunction by a mechanism involving suppression of MYD88/NF-κb pathway and inflammation.

## Introduction

Obesity is becoming an epidemic in worldwide due to increasingly sedentary lifestyles, over-nutrition and an escalating aging population. Heart disease is increased by up to ten-fold in people with obesity compared to the general age-matched population. Obesity is associated with significantly increased mortality and morbidity due to type 2 diabetes and cardiovascular diseases. Obesity-induced cardiomyopathy is characterized by abnormal cardiac morphology and function, independent of vascular complications. Although obesity is well-known independent risk factors for cardiovascular diseases and cardiomyopathy, the molecular mechanisms that link obesity to cardiac dysfunction and cardiomyopathy are poorly understood.

Hypoxia inducible factor (HIF) is a heterodimeric protein consisting of two subunits: HIF-α (HIF-1α, -2α and -3α) and HIF-β [1]. HIF-α plays a central role in the transcriptional response to changes in oxygen availability. HIF-α stabilization is regulated by the prolyl hydroxylases (PHDs) family, the main oxygen sensors which target HIF-α for ubiquitination and proteasomal degradation [1]–[3]. In humans, the PHD family is composed of three different isoforms PHD1, PHD2 and PHD3, all of which requires iron and ascorbate as cofactors [4]. Treatment with PHD inhibitors has been shown to reduce myocardial fibrosis after myocardial infarction in rats. This is accompanied by a significant improvement in cardiac function [5]. Most recent studies also suggest that PHDs play important roles in the regulation of glucose and fatty acid metabolism [6]–[9]. Knockout of PHD2 in adipocytes reduces HFD-induced obesity and improves glucose tolerance in a HIF-1α-dependent manner [6]. Moreover, knockout of PHD3 enhances insulin sensitivity in diabetes mellitus by stabilizing HIF-2α [8]. Accumulating evidence suggests that PHDs are responding to stimuli other than oxygen, and HIF-α is not the sole PHDs effector [10]–[12]. For example, PHDs have been shown to negatively regulate nuclear factor-kappa B (NF-κb) activity by inhibition of NF-κB kinase-β hydroxylation [10]. Pharmacological inhibition of PHDs suppresses lipopolysaccharide-induced TNFα induction by reducing NFκB transcriptional activity [13]. Activation of NF-κb has been shown to be associated with HFD–induced vascular inflammation and diabetic cardiomyopathy [14]–[18]. A recent study also indicates a critical role of PHDs in the regulation of innate immunity and inflammation [12]. Nevertheless, the functional role of PHD2 in obesity associated cardiomyopathy has not previously been examined. In particular, it is unclear whether specific blockade of PHD2 could prevent or reverse HFD-induced cardiac dysfunction.

In the present study, we hypothesized that PHD2 activity contributes to HFD-induced cardiomyopathy. Using PHD2 knockout mice fed a HFD, we investigated the effects of PHD2 on the development of cardiomyopathy. We have demonstrated that elevation of PHD2 activity in the heart contributed to HFD-induced cardiac inflammation and dysfunction. The beneficial effects of PHD2 inhibition were through mechanisms involving suppression of MYD88/NF-κb-65 signaling and improvement of glucose tolerance.

## Materials and Methods

### Ethic Statement

The investigation conforms to the Guide for the Care and Use of Laboratory Animals published by the US National Institutes of Health (NIH Publication No. 85-23, revised 1996). The protocol was approved by the Committee on the Ethics of Animal Experiments of the University of Mississippi Medical Center (Protocol ID: 1280). The experimental mice were anesthetized with ketamine.

### Animal studies and diet-induced obesity

The C57BL/6J male (wild type, WT) at age of 8 weeks were purchased from the Jackson laboratory (Bar Harbor, ME). The WT mice were placed with either a normal chow diet or a high-fat diet (D12492 60% kcal diet, Research Diets Inc, NJ) for 16 weeks to produce a diet-induced obesity model. The PHD2^flox/flox^ mice were originally provided by Dr. Guo-fan Fong at University of Connecticut Medical Center. The PHD2^flox/flox^ mouse was crossed with B6-ROSA-Cre/ERT2 (WT-Cre^+^) to generate a PHD2^f/f^-Cre^+^ [19]. WT-Cre^+^, PHD2^f/f^-Cre^+^ and PHD2^f/−^Cre^+^ mice were bred by our colonies. Male WT-Cre^+^, PHD2^f/−^Cre^+^ and PHD2^f/f^-Cre^+^ mice at age of 8 weeks were administrated with tamoxifen (1 mg/day in corn oil, Sigma, MO) for 7 days to deletion PHD2 before fed chow diet or HFD. The deletion of PHD2 gene was confirmed by western blot analysis. Two weeks after tamoxifen administration, the experimental mice were then fed either a normal chow diet or a high-fat diet (D12492 60% kcal diet, Research Diets Inc, NJ) for 16 weeks. Body weight and glucose levels were monitored every 2 to 4 weeks interval [20].

### Western analysis of PHD1-3, HIF-1α, Tie-2, VEGF, Ang-1, Ang-2, TLR4, NFκbp65, MyD88, TNFα, IRAK-4 and ICAM-1 expression

The hearts were harvested and homogenized in lysis buffer for Western blot analysis. Following immunoblotting, the membranes were immunoblotted with VEGF, Ang-2, Tie-2, NFκbp65, ICAM-1, MyD88, IRK-4, TNFα and TLR4 (1∶1000, Santa Cruz, CA), PHD1, PHD2, PHD3 and HIF-1α (Novus Bio, CO) and Ang-1 (1∶1000, Sigma, MO) antibodies. The membranes were then washed and incubated with a secondary antibody coupled to horseradish peroxidase and densitometric analysis was carried out using image acquisition and analysis software (TINA 2.0). Cardiac hypertrophic gene β-myosin heavy chain (β-MHC) and ANP expression was examined by western blot analysis. Heart tissue sections were stained with H&E (Haematoxylin and Eosin, Sigma, MO). Cardiomyocyte size (area) (40X) was measured by using NIH image analysis.

### Myocardial apoptosis and macrophage infiltration

Heart tissue sections were stained with transferase deoxyuridine nick end labeling (TUNEL) following the manufacturer’s instructions (Promega, WI). Apoptosis was identified as TUNEL positive cells. The infiltration of macrophage in the heart tissues was assessed by stained with CD11b and CD45 antibodies.

### Echocardiography

Transthoracic two-dimensional M-mode echocardiography was performed using a Visual Sonics Vevo 770 Imaging System (Toronto, Canada) equipped with a 707B high frequency linear transducer. Mice were anesthetized using a mixture of isoflurane (1.5%) and oxygen (0.5 L/min). The short-axis imaging was taken as M-mode acquisition for 30 seconds. End-systolic and end-diastolic dimensions, end-systolic and end-diastolic volumes, stroke volume were recorded to calculate the percent fractional shortening (%FS) and ejection fractions (EF%). Data analysis was performed with the use of a customized version of Vevo 770 Analytic Software [21].

### Hemodynamic measurements

The experimental mice were anesthetized with ketamine (100 mg/kg) plus xylazine (15 mg/kg), intubated and artificially ventilated with room air. A 1.4-Fr pressure–conductance catheter (SPR-839, Millar Instrument, TX) was inserted into the left ventricle (LV) to record baseline cardiac hemodynamics of the hearts. Raw conductance volumes were corrected for parallel conductance by the hypertonic saline dilution method [22].

### Glucose tolerance test

After 16 weeks of HFD, the experimental mice were subjected to glucose tolerance test using the procedure described previously [6], [8]. Glucose tolerance test was carried out after a 12 hour fast by intraperitoneal injection with D-glucose (1 mg/g) in sterile saline. Blood was obtained from experimental mice by tail snip, and blood glucose levels were measured with One Touch SureStep test strips. Glucose levels were expressed as mg/dL.

### Statistical analysis

The results were expressed as the mean ± SD. Statistical analysis was performed using one way ANOVA followed by Student t-test. Significance was set at *P*<0.05.

## Results

### 1. PHD2 expression is enhanced in the hearts of HFD mice

**Figure 1:**
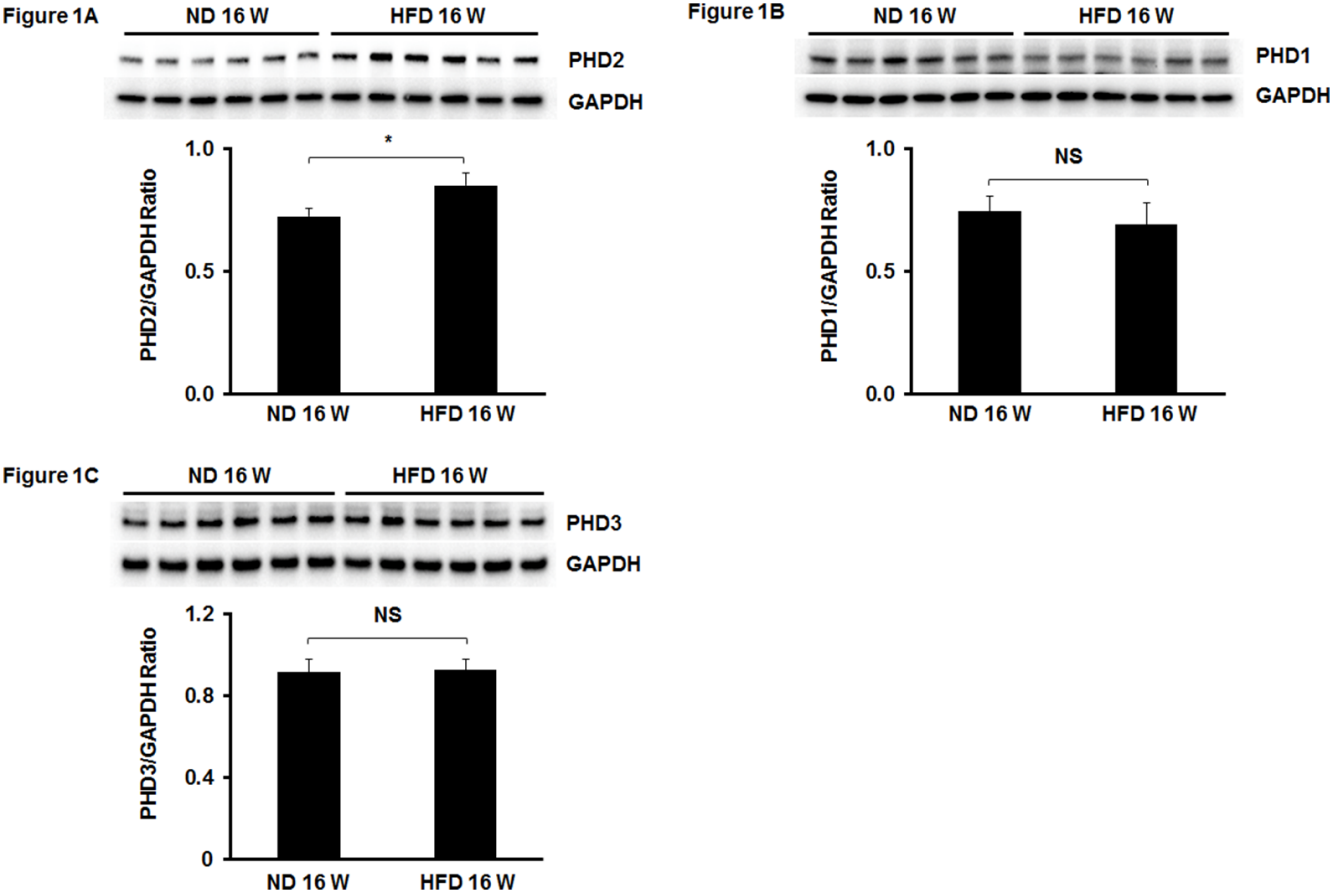
Expression of PHDs in the hears of HFD mice. (A) Western blot analysis demonstrating that PHD2 expression was significantly upregulated in the hearts of HFD mice compared to normal chow diet (ND) mice (n = 6 mice, *p<0.05). (B and C) Western blot analysis of PHD1 and PHD3 expression showing that there was no significantly difference in PHD1 and PHD3 expression between HFD and normal diet (ND) mice (n = 6 mice, p>0.05). NS =  Not significant.

**Figure 2:**
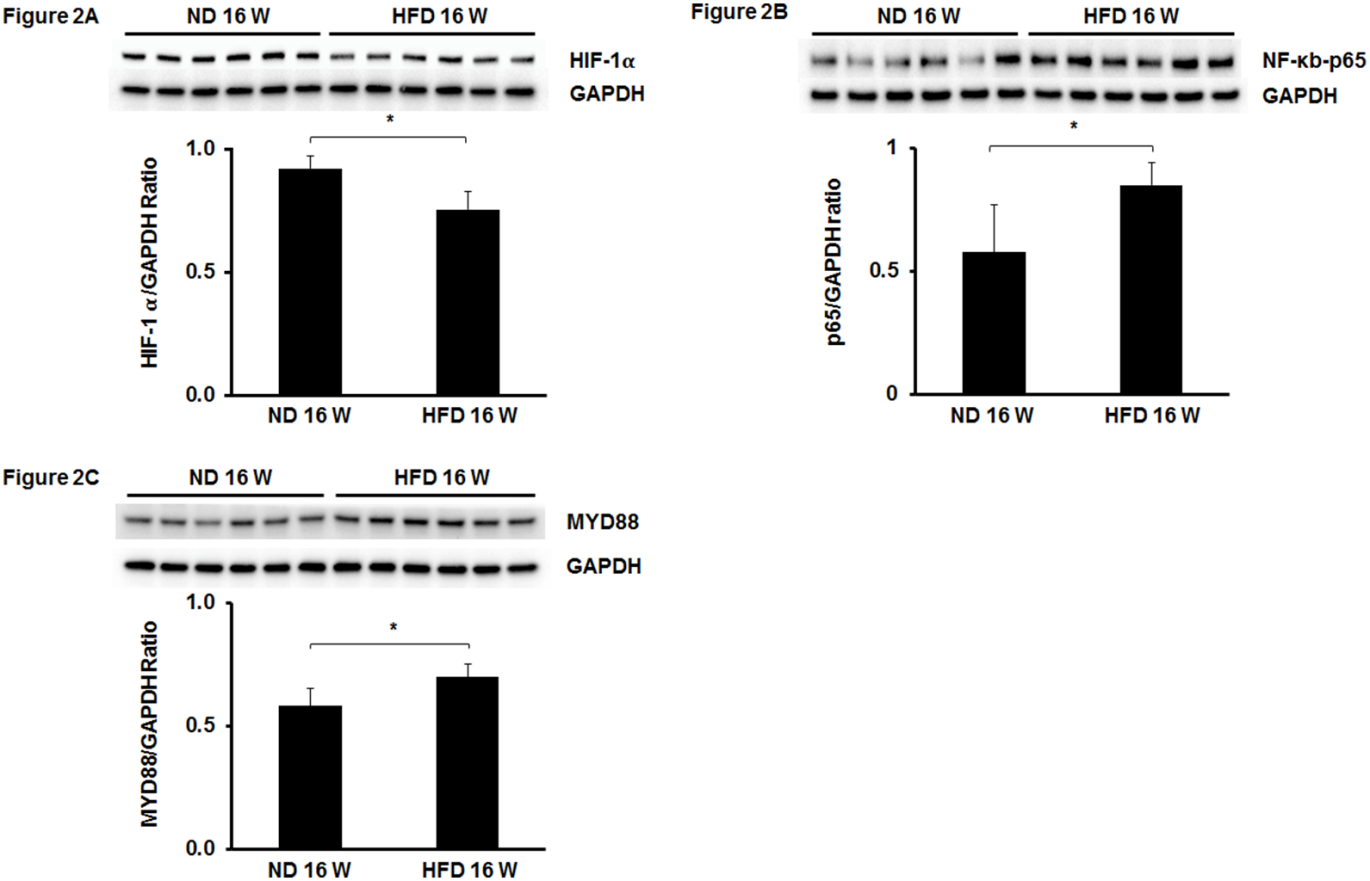
Expression of HIF-1α, NF-κb and MyD88 in the hears of HFD mice. (A and B) Western blot analysis revealing that HIF-1α expression was significantly decreased in the hearts of HFD mice compared to normal diet (ND) mice (n = 6 mice, *p<0.05). (B) NF-κb expression was significantly upregulated in the hearts of HFD mice compared to normal diet (ND) mice (n = 6 mice, *p<0.05). (C) MyD88 expression was significantly increased in the hearts of HFD mice compared to normal diet (ND) mice (n = 6 mice, *p<0.05).

We first examined PHD1-3 expression in the hearts of HFD mice. Mice fed a HFD for 16 weeks led to a significant increase in PHD2 expression when compared to normal chow diet (ND) fed mice (Fig. 1A). In contrast, HFD fed mice had little effect on the expression of PHD1 and PHD3 in the heart (Fig. 1B and C). To further confirm activation of PHD2 in HFD mouse hearts, we examined PHD2 target genes HIF-α and NF-κb p65 expression. The expression of HIF-1α was significantly reduced in the hearts of HFD mice (Fig. 2A). Interestingly, NF-κb p65 expression was significantly increased in the hearts of HFD mice (Fig. 2B). Since MYD88 has been shown to be involved in NF-κb p65 activation and cardiac hypertrophy [23], [24], we measured MYD88 expression in HFD fed mice. We found that MYD88 expression was significantly increased in the hearts of HFD mice when compared with ND mice (Fig. 2C).

### 2. Mice fed a HFD leads to an impairment of cardiac function

**Figure 3:**
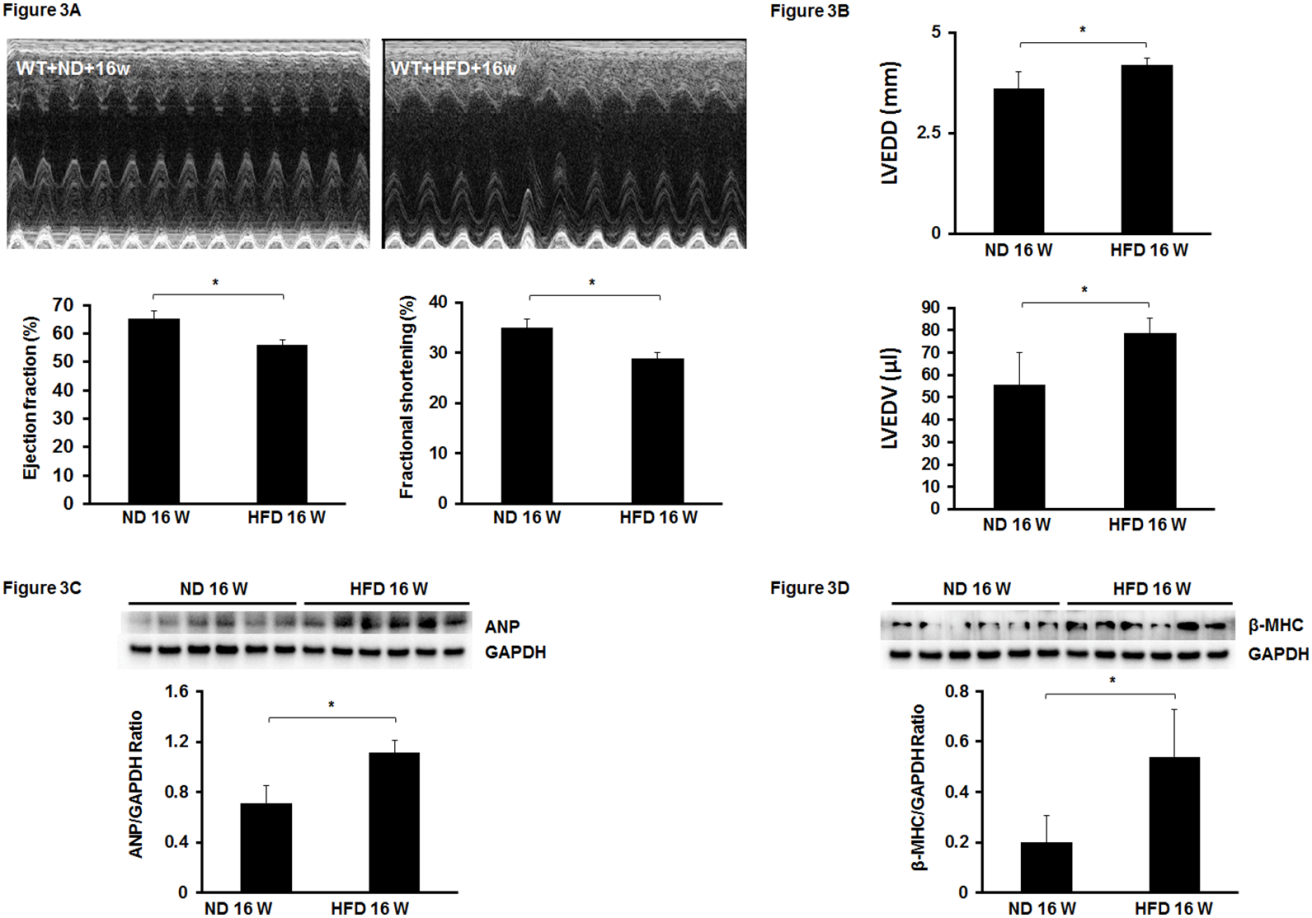
Feeding with HFD for 16 weeks leads to cardiac dysfunction in mice. (A) Upper panel: Representative images of M mode of echocardiography. Low panel: Feeding mice with HFD for 16 weeks significantly impaired cardiac performance indicated by decreases in EF and FS levels in HFD mice compared to ND mice (n = 5–7 mice, *p<0.05). (B) Left ventricular end diastolic diameter (LVEDD) and left ventricular end diastolic volume (LVEDV) were significantly elevated in HFD mice compared to ND mice at 16 weeks (n = 5–7 mice, *p<0.05). (C and D) Expression of cardiac hypertrophic markers ANP and β-MHC in HFD and ND mice. The levels of ANP and β-MHC were significantly higher in the hearts of HFD mice than that of ND mice at 16 weeks (n = 6 mice, *p<0.05).

Next, we examined whether mice fed a HFD for 16 weeks caused cardiac dysfunction. As expected, mice on HFD had an impaired cardiac function and exhibited a significant reduction of ejection fraction (EF%) and ejection shortening (FS%) when compared with mice on ND (Fig. 3A). Moreover, LVEDD and LVEDV levels were significantly elevated in HFD fed mice compared to ND fed mice (Fig. 3B). In addition, cardiac hypertrophic marker β-MHC and ANP expression was significantly increased in the hearts of HFD fed mice (Fig. 3C and D). These results confirmed the development of cardiomyopathy in mice on HFD.

### 3. Knockout of PHD2 upregulates HIF-1α and reduces glucose levels in HFD mice

**Figure 4:**
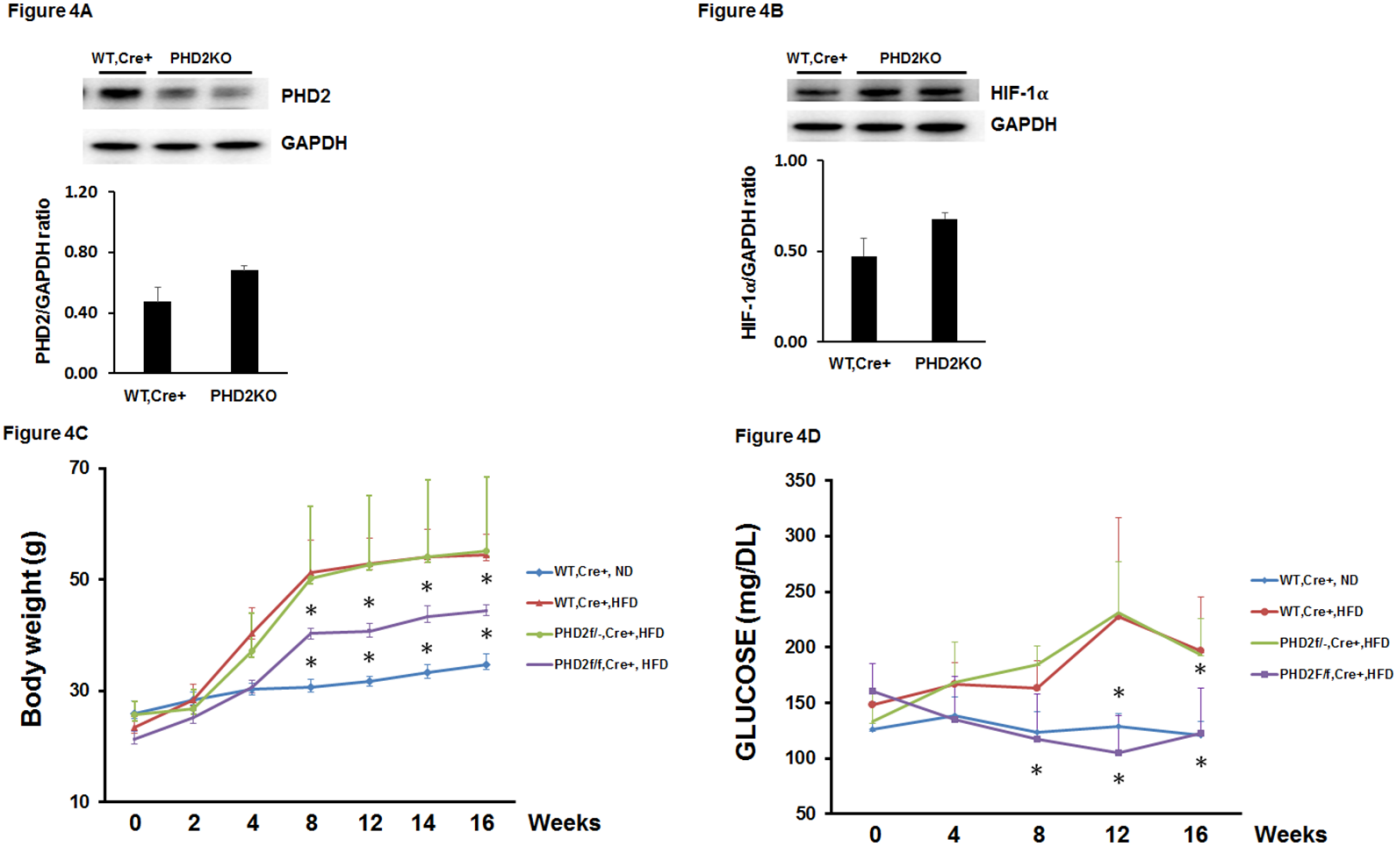
Knockout of PHD2 prevents HFD-induced increases in body weight and glucose levels in mice. (A and B) Western blot analysis confirming that treated PHD2^f/f-Cre+^ mice with tamoxifen for 7 days reduced PHD2 expression in the hearts. The expression of HIF-1α was increased in the hearts of PHD2^f/f-Cre+^ mice treated with tamoxifen for 7 days compared to wild type (WT, Cre^+^) mice treated with tamoxifen for 7 days (n = 2 mice). (C and D) Pretreatment of WT, Cre^+^ mice with tamoxifen for 7 days then fed with HFD for 16 weeks led to a gradual increase in body weight and elevation of fasting glucose levels compared to WT, Cre^+^ mice fed with normal chow diet (ND) (n = 10 mice, *p<0.05). Pretreatment of PHD2^f/f-Cre+^ mice with tamoxifen for 7 days then fed with HFD for 16 weeks significantly reduced body weight growth and fasting glucose levels compared to WT, Cre^+^ mice fed with HFD (n = 10 mice, *p<0.05). Pretreatment of PHD2^f/−Cre+^ mice with tamoxifen for 7 days then fed with HFD for 16 weeks did not alter body weight growth and fasting glucose levels compared to WT, Cre^+^ mice fed with HFD (n = 10 mice, p>0.05).

To examine the role of PHD2 in diabetic cardiomyopathy, we used PHD2 knockout mice (PHD2KO) fed a HFD for 16 weeks. PHD2^f/f^-Cre^+^ mice at 8 weeks age were administrated with tamoxifen for 7 days to knockout of PHD2 protein. Consistent with previous study [25], treatment of PHD2^f/f^-Cre^+^ mice with tamoxifen for 7 days led to 50% decrease in PHD2 expression in the heart (**Fig. 4A**). This was accompanied by a two-fold increase in HIF-1α expression in the hearts of PHD2KO mice (**Fig. 4B**). The PHD2KO mice were then fed a HFD for 16 weeks. Feeding WT-Cre^+^ mice HFD resulted in a gradual increase in body weight growth during 16 weeks of study. Body weight growth was significantly less in PHD2KO mice than WT-Cre^+^ mice on HFD (**Fig. 4C**). WT-Cre^+^ mice fed a HFD for 16 weeks resulted in a gradual increase in fasting blood glucose levels. Interestingly, the fasting glucose levels were significantly reduced in PHD2KO mice when compared with WT-Cre^+^ mice on HFD (**Fig. 4D**). PHD2^f/−^Cre^+^ mice had little effects on HFD-induced body weight growth and fasting glucose levels (**Fig. 4C and D**).

### 4. Knockout of PHD2 attenuates HFD-induced apoptosis and cardiac dysfunction

**Figure 5:**
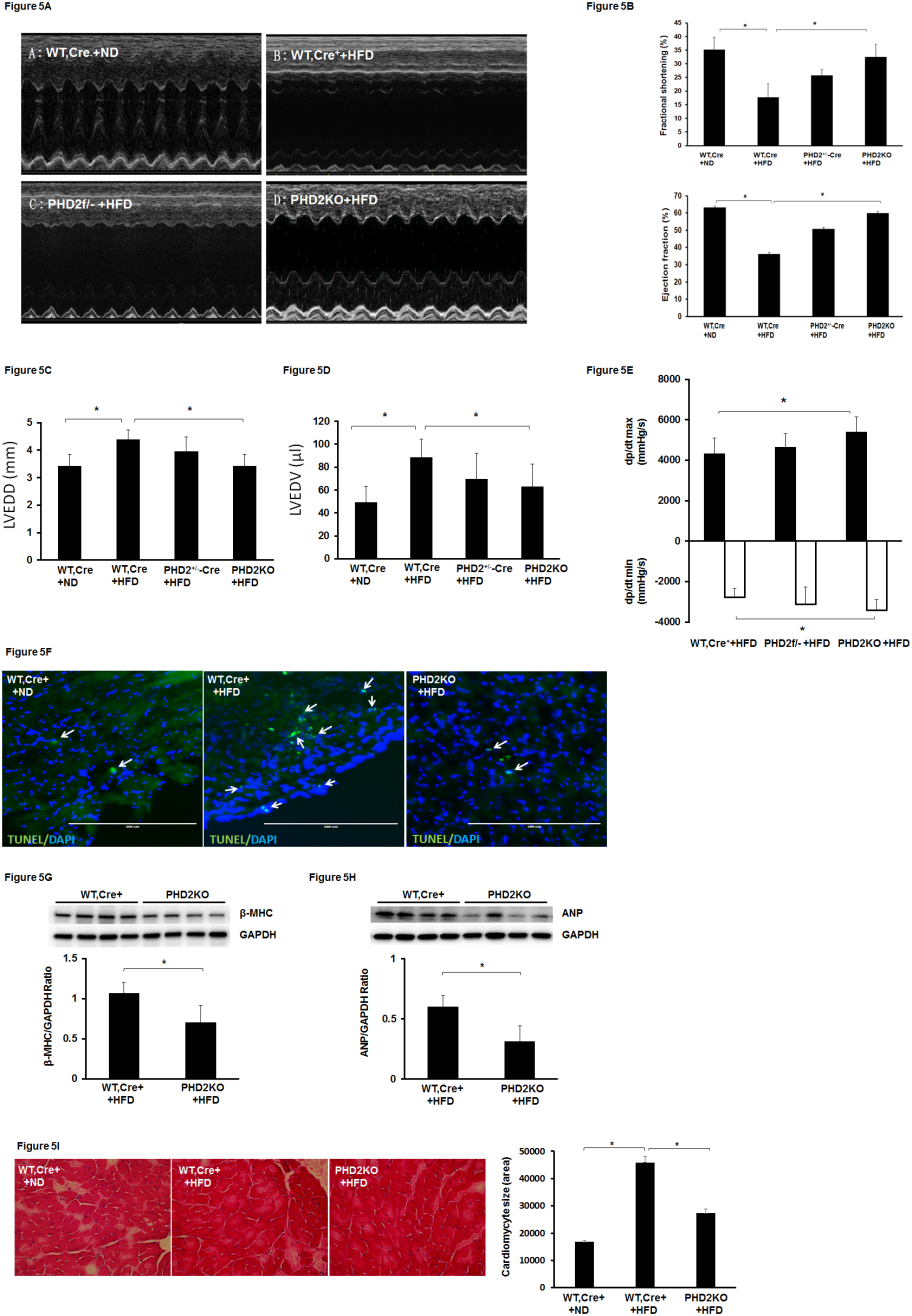
Knockout of PHD2 improves HFD-induced impairment of cardiac function in mice. (A) Representative images of M mode of echocardiography. (B) Pretreatment of WT, Cre^+^ mice with tamoxifen for 7 days then fed with HFD for 16 weeks resulted in a significant reduction of FS% and EF% compared to WT, Cre^+^ mice fed with ND (n = 7 mice, *p<0.05). Pretreatment of PHD2^f/f-Cre+^ mice with tamoxifen for 7 days then fed with HFD for 16 weeks significantly increased FS% and EF% levels compared to WT, Cre^+^ mice fed with HFD (n = 7 mice, *p<0.05). Pretreatment of PHD2^f/−Cre+^ mice with tamoxifen for 7 days then fed with HFD for 16 weeks had little effects on FS% and EF% levels compared to WT, Cre^+^ mice fed with HFD (n = 6 mice, p>0.05). (C and D) Left ventricular end diastolic diameter (LVEDD) and left ventricular end diastolic volume (LVEDV) were significantly elevated in WT, Cre^+^ + HFD mice compared to WT, Cre^+^ + ND mice at 16 weeks (n = 5–7 mice, *p<0.05). Knockout of PHD2 (PHD2KO) significantly reduced HFD-induced elevation of LVEDD and LVEDV (n = 7 mice, *p<0.05). (E) Cardiac dp/dt max and dp/dt min levels were significantly improved in PHD2KO + HFD mice compared to WT, Cre^+^ + HFD mice (n = 6 mice, *p<0.05). (F) Apoptotic cells were stained with apoptotic marker TUNEL (green) and nuclei were counterstained with DAPI (blue). Immunohistochemical analysis of apoptotic cells showing apoptotic cells (TUNEL positive cells, white arrow) were increased in the hearts of WT, Cre^+^ + HFD mice compared to that of WT, Cre^+^ + ND mice. Knockout of PHD2 dramatic reduced the number of TUNEL positive cells in the hearts of HFD mice. (G and H) The levels of ANP and β-MHC were significantly reduced in the hearts of PHD2KO + HFD mice compared to WT, Cre^+^ + HFD mice at 16 weeks (n = 4–5 mice, *p<0.05). (I) Cardiomyocytes were stained with H&E. Cardiomyocyte size (area) was significantly reduced in the hearts of PHD2KO + HFD mice compared to WT, Cre^+^ + HFD mice at 16 weeks (40X, n = 3 mice, *p<0.05).

WT-Cre^+^ mice fed a HFD for 16 weeks led to a gradual decline in cardiac function. The echocardiography analysis showed that the basal levels of ejection fraction (EF%) and fractional shortening (FS%) were significantly reduced in HFD fed WT-Cre^+^ mice when compared with ND fed WT-Cre^+^ mice (**Fig. 5A and B**). The basal levels of EF (%) and FS (%) were significantly elevated in PHD2KO mice compared with WT-Cre^+^ mice on HFD (**Fig. 5A and B**). Moreover, LVEDD and LVEDV levels were significantly reduced in PHD2KO mice compared to WT-Cre^+^ mice fed a HFD (**Fig. 5C and D**). The changes in dp/dtmax and dp/dtmin were also significantly improved in PHD2KO mice compared with WT-Cre^+^ mice on HFD (**Fig. 5E**). Although the EF% and FS% were increased in HFD fed PHD2^f/−^Cre^+^ mice, these changes did not reach significance. WT-Cre^+^ mice fed HFD for 16 weeks resulted in an increase in apoptosis in the heart. The number of apoptotic cells was dramatic reduced in HFD fed PHD2KO mice when compared with HFD fed WT-Cre^+^ mice **(** **Fig. 5F** **)**. In addition, cardiac hypertrophy markers β-MHC and ANP expression were significantly reduced in the hearts of HFD fed PHD2KO mice (**Fig. 5G and H**). WT-Cre^+^ mice fed HFD for 16 weeks resulted in a significant increase in cardiomyocyte size. Cardiomyocyte size was significantly reduced in HFD fed PHD2KO mice when compared with HFD fed WT-Cre^+^ mice (**Fig. 5I** **)**.

### 5. Knockout of PHD2 does not increase HIF-1α and angiogenic growth factors in the hearts of HFD mice

**Figure 6:**
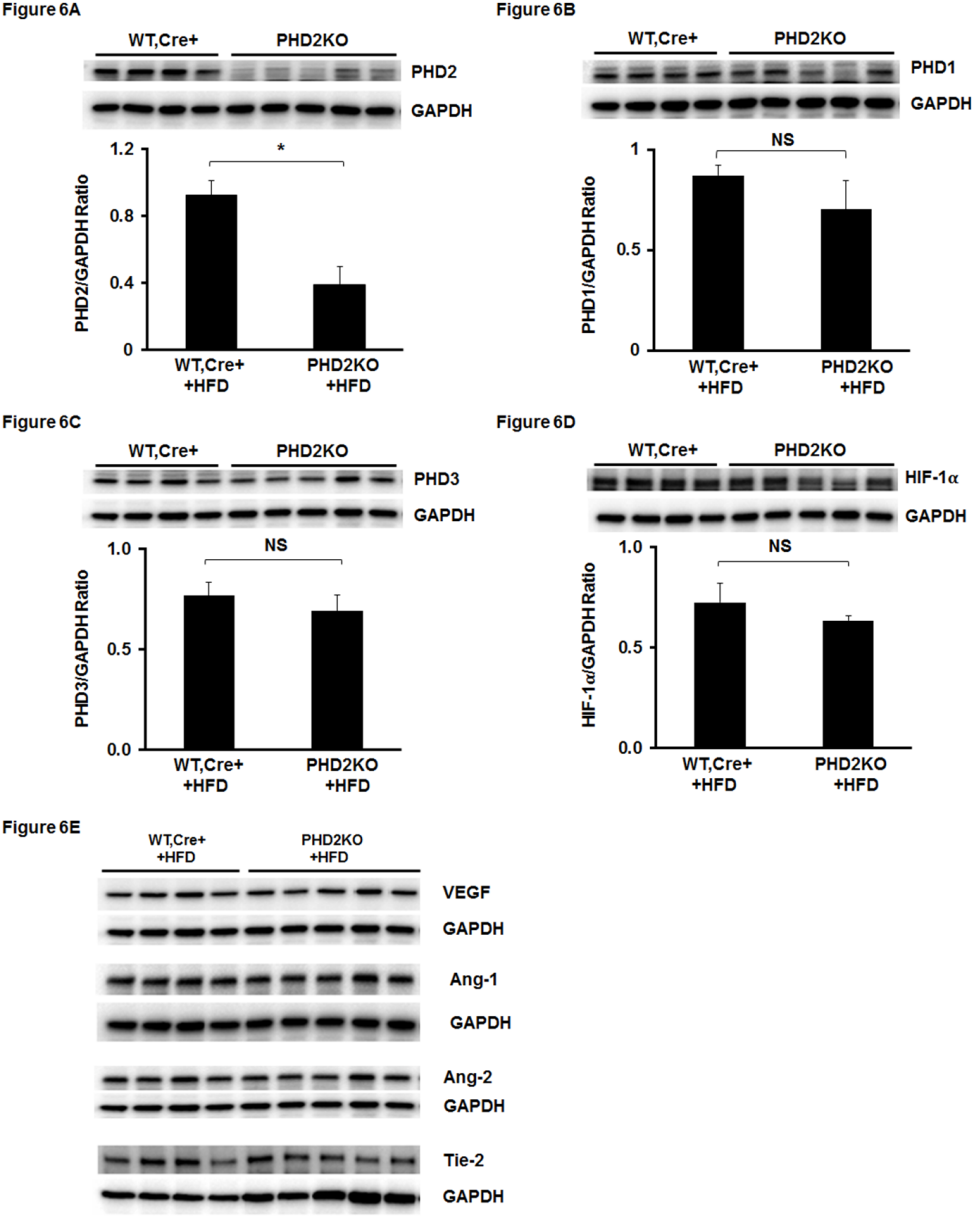
Knockout of PHD2 does not rescue HFD-induced reduction of HIF-1α expression in HFD mice. (A) Western blot analysis confirming that PHD2 expression was significantly reduced in PHD2^f/f-Cre+^ mice treated with tamoxifen for 7 days and fed with HFD for 16 weeks compared to WT, Cre^+^ + HFD mice (n = 4–5 mice, *p<0.05). (B–F) Western blot analysis showing that expression of PHD1 and PHD3 was not significantly altered in PHD2KO + HFD mice compared to WT, Cre^+^ + HFD mice (n = 4–5 mice, NS). The expression of HIF-1α did not changed in the hearts of PHD2KO + HFD mice compared to WT, Cre^+^ + HFD mice (n = 4–5 mice, NS). VEGF and angiopoietins/Tie-2 system were not altered in PHD2KO + HFD mice compared to WT, Cre^+^ + HFD mice (n = 4–5 mice, NS).

To explore the potential molecular mechanisms by which knockout of PHD2 attenuated cardiac dysfunction in HFD mice, we examined HIF-1α and its downstream target gene expression. As shown in Fig. 6A, PHD2 expression was significantly reduced in the hearts of HFD fed PHD2KO mice compared to HFD fed WT-Cre^+^ mice (**Fig. 6A**). The expression of PHD1 and PHD3 remained unchanged in the hearts of HFD fed PHD2KO mice (**Fig. 6B and C**). Furthermore, knockout of PHD2 in mice had little effect on the expression of HIF-1α in the heart (**Fig. 6D and E**). In addition, HIF-1α downstream gene VEGF, Ang-1, Ang-2 and Tie-2 expression was not altered in the hearts of HFD fed PHD2KO mice (**Fig. 6E**).

### 6. Knockout of PHD2 attenuates NFκb-p65 expression and macrophage infiltration in the hearts of HFD mice

**Figure 7:**
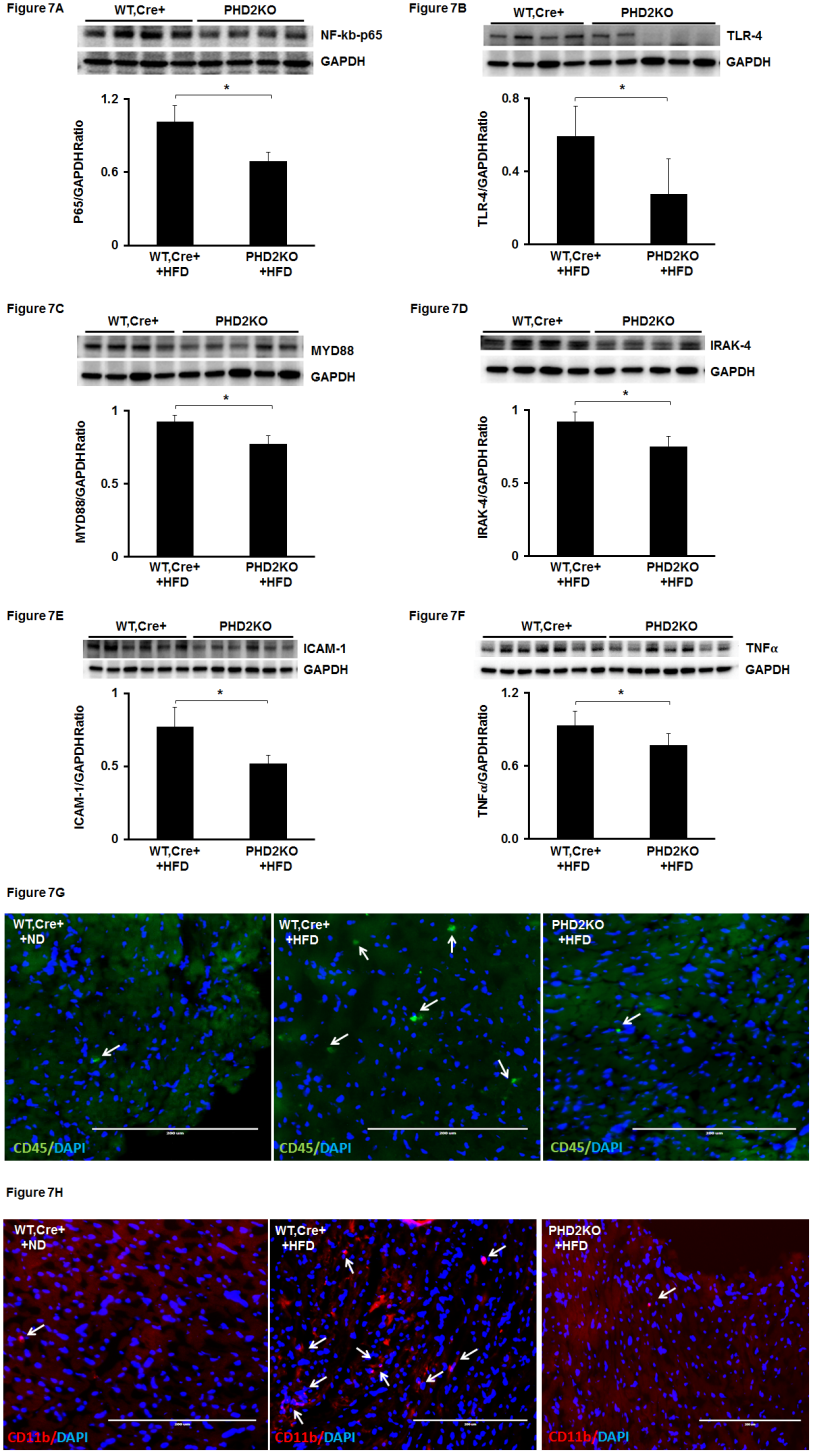
Knockout of PHD2 reduces HFD-induced NF-κb activation and macrophage infiltration in HFD mice. (A) NF-κb expression was significantly decreased in the hearts of PHD2KO+HFD mice compared to WT, Cre^+^ + HFD mice at 16 weeks (n = 4 mice, *p<0.05). (B) TLR4 expression was significantly reduced in the hearts of PHD2KO+HFD mice compared to WT, Cre^+^ + HFD mice at 16 weeks (n = 4–5 mice, *p<0.05). (C) MyD88 expression was significantly suppressed in the hearts of PHD2KO+HFD mice compared to WT, Cre^+^ + HFD mice at 16 weeks (n = 4–5 mice, *p<0.05). (D) IRAK-4 expression was significantly inhibited in the hearts of PHD2KO+HFD mice compared to WT, Cre^+^ + HFD mice at 16 weeks (n = 4 mice, *p<0.05). (E and F) The expression of ICAM-1 and TNF-α was significantly reduced in the hearts of PHD2KO+HFD mice compared to WT, Cre^+^ + HFD mice at 16 weeks (n = 6 mice, *p<0.05). (G and H) Inflammatory cell infiltrations were stained with macrophage markers CD45 (green) and CD11b (Red). Nuclei were counterstained with DAPI (blue). Immunohistochemical analysis of macrophage infiltrations (CD45 and CD11b positive cells) showing the number of macrophage (white arrow) was increased in the hearts of WT, Cre^+^ + HFD mice compared to that of WT, Cre^+^ + ND mice. Knockout of PHD2 dramatic reduced the number of macrophage in the hearts of HFD mice.

We then examined whether knockout of PHD2 reduced HFD-induced NFκb p65 expression in the heart. Knockout of PHD2 led to a significant decrease in NFκb-p65 expression compared with WT-Cre^+^ mice on HFD (**Fig. 7A**). TLR4/MYD88 has been shown to have a critical role in NF-κb-mediated cardiac hypertrophy and inflammation. We further examined whether inhibition of PHD2 reduced TLR4/MYD88 expression and inflammation in the hearts of HFD mice. The expression of TLR4 and MyD88 was significantly reduced in the hearts of PHD2KO mice compared with that of WT-Cre^+^ mice (**Fig. 7B and C**). IRAK-4, TNFα and ICAM-1 expression was also significantly suppressed in PHD2KO mice compared to WT-Cre^+^ mice (**Fig. 7D, E and F**). To examine infiltration of macrophages in the heart tissue, we used immunohistochemical staining for CD11b and CD45. The number of infiltration of macrophages was dramatic less in PHD2KO mice than WT-Cre^+^ mice (**Fig. 7G and H**).

### 7. Conditional knockout of PHD2 at late stage in obese mice improves glucose tolerance and reverses impaired cardiac function

**Figure 8:**
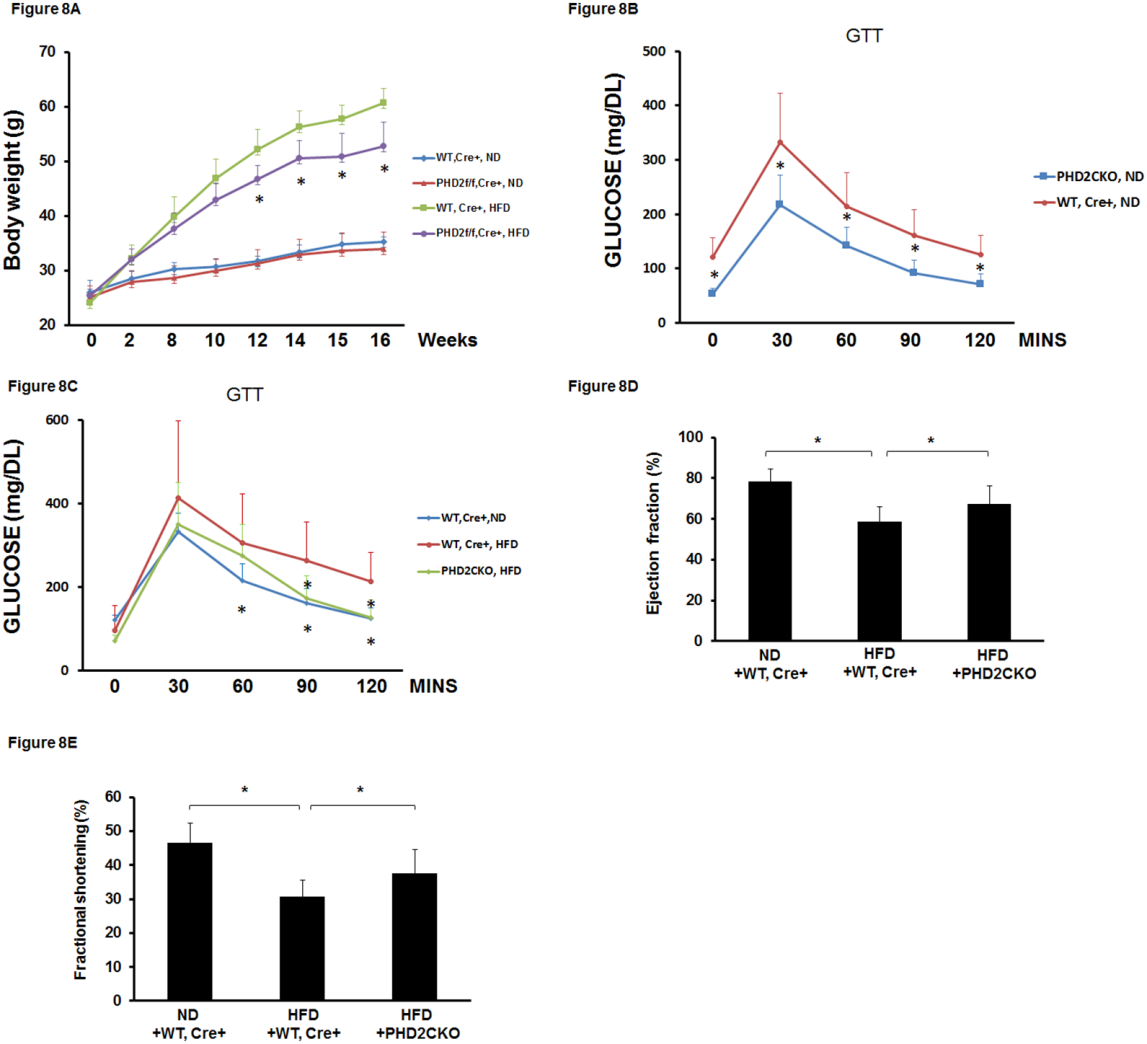
Conditional knockout of PHD2 in HFD mice improves glucose tolerance and cardiac function. (A) Conditional knockout of PHD2 in HFD mice significantly reduced body weight growth compared to WT, Cre^+^−HFD mice (n = 10 mice, *p<0.05). (B) Conditional knockout of PHD2 in normal chow diet mice significantly improved glucose tolerance compared to WT, Cre^+^ mice fed with ND (n = 7–10 mice, *p<0.05). (C) Conditional knockout of PHD2 in HFD mice significantly enhanced glucose tolerance compared to WT, Cre^+^−HFD mice (n = 10 mice, *p<0.05). (D and E) Conditional knockout of PHD2 in HFD mice significantly increased EF% and FS% compared to WT, Cre^+^−HFD mice (n = 5–7 mice, *p<0.05).

To test whether conditional knockout of PHD2 at late stage of obesity could reverse impaired cardiac function, PHD2^f/f^-Cre^+^ mice were fed a HFD for 12 weeks to induce obesity and then were administrated with tamoxifen for 7 days to delete PHD2. Glucose tolerance and cardiac function were assessed after 4 weeks of PHD2 deletion. There was no significant difference in the body weight gain between PHD2CKO mice and WT-Cre^+^ mice on ND. Conditional knockout of PHD2 in obese mice resulted in a significant loss of body weight when compared with WT-Cre^+^ obese mice (**Fig. 8A**). Glucose tolerance test showed that glucose tolerance was significantly improved in PHD2CKO mice when compared with WT-Cre^+^ mice on ND (**Fig. 8B**). Similarly, glucose tolerance was significantly improved in PHD2CKO obese mice compared to WT-Cre^+^ obese mice (**Fig. 8C**). As shown in **Fig. 8D and E**, conditional knockout of PHD2 in obese mice significantly increased EF% and FS% levels compared with WT-Cre^+^ obese mice.

## Discussion

In the present study, we have demonstrated that PHD2 activity was a critical contributor to HFD-induced cardiac dysfunction in mice. Our data showed that the PHD2 levels were persistently elevated in the hearts of HFD fed mice. This was accompanied by a significant upregulation of NF-κb p65 and downregulation of HIF-1α expression. Knockout of PHD2 in mice prevented HFD-induced cardiac dysfunction. Moreover, conditional knockout of PHD2 in obese mice reversed cardiac dysfunction. Mechanistically, knockout of PHD2 led to a significant suppression of myocardial MYD88/NF-κb p65 expression in obese mice. Most intriguingly, conditional knockout of PHD2 at late stage of obesity significantly reduced fasting glucose level and dramatic improved glucose tolerance. Our data suggested inhibition of PHD2 attenuated HFD-induced cardiac dysfunction by a mechanism involving suppression of MYD88/NFκb and improvement of glucose tolerance.

Obesity-associated cardiomyopathy is independent of hypertension and coronary diseases. Although extensive studies have been done, the signaling events and molecular mechanisms by which HFD causes heart dysfunction have not been well understood. To our knowledge, our study was the first to provide direct evidence that elevated PHD2 played a critical role in HFD-mediated cardiac dysfunction. We found that PHD2 expression was significantly increased in the hearts of mice on HFD. Our data also revealed that HIF-1α expression was impaired in HFD fed mice. HIF-1α is the main transcription factor that controls cellular metabolism in the heart. PHD2 has been shown to reduce HIF-1α levels by ubiquitination and proteasomal degradation [1]–[3]. Previously we reported that PHD2 levels were elevated in the hearts of obese diabetic db/db mice. We also demonstrated that suppression of PHD2 was associated with reduction of cardiac hypertrophy and fibrosis [26]. So far, the direct links between PHD2 and HFD-induced cardiac dysfunction have not been fully investigated. The present study extended our previous findings and investigated whether knockout of PHD2 prevented HFD-induced cardiomyopathy. Therapeutically, we examined whether conditional knockout of PHD2 rescued impaired cardiac function in obesity. Our data clearly showed that knockout of PHD2 prevented the development of HFD-induced cardiac dysfunction. Moreover, conditional knockout of PHD2 rescued impaired cardiac function at late stage of obesity.

HIF-α regulates many metabolic pathways and has a critical role in the regulation of cellular metabolisms. Recent studies highlight the importance of PHD2 in the regulation of glucose and lipid metabolism [6]–[9]. Using PHD2^f/f^-aP2-Cre mice, it has been shown that deletion of PHD2 in adipocytes attenuates high-fat diet-induced obesity. Knockout of PHD2 in adipocytes significantly increases HIF-1α and adiponectin expression [6]. Consistent with this study, we also showed that knockout of PHD2 increased HIF-1α expression and reduced HFD-induced obesity. Most interestingly, conditional knockout of PHD2 at late stage of obesity completely reversed glucose tolerance. Taken together, these data suggest that specific inhibition of PHD2 may be a novel target for obesity-associated glucose or insulin resistance. Furthermore, PHD2 inhibition mediated improvements of body weight and glucose tolerance may be attributed, at least in part, to cardiac function enhancement in HFD mice. While we were doing our study, another group reported that activation of HIF-2α in adipocytes resulted in cardiac hypertrophy. This study suggested that elevation of HIF-2α levels in the normal heart was detrimental [27]. However, in obese hearts, the picture was quite different because the expression of HIF-1*α* was significantly decreased compared to non-obese heart. Therefore, restoration of impaired HIF-1*α* was protective and beneficial in the obese heart.

Chronic inflammation plays a critical role in promoting diabetes-associated cardiovascular complications [16], [17]. Chronic inflammation is a hallmark of heart failure [28]–[30]. Transcription factor NF-κb regulates genes that induce inflammation as well as apoptosis and hypertrophy in cardiomyocytes [31]–[33]. NF-κb activity is increased in many heart diseases such as congestive heart failure, myocardial hypertrophy and ischemic reperfusion [34]–[37]. Activation of NF-κb is also involved in diabetes-associated oxidative stress, inflammation and endothelial dysfunction [16]–[18]. In particular, NF-κb activation in the heart may contribute to diabetic cardiomyopathy. It has been reported that hyperglycemia stimulates myocardial NF-κb activation in diabetic cardiomyopathy [14], [15]. A recent study shows that persistent NF-κB p65 activation promotes pro-inflammatory, pro-fibrotic and pro-apoptotic effects, and exacerbates cardiac hypertrophy and apoptosis in heart failure [32]. Consistent with these findings, we showed that mice fed a HFD increased NF-κb p65 expression in the heart. This was accompanied by a dramatic decline in cardiac function. Moreover, knockout of PHD2 significantly inhibited NF-κb p65 expression together with a dramatic reduction of apoptosis in the hearts of HFD mice. Our data suggest a potential novel PHD2-NF-κb p65 axis in HFD-induced cardiomyopathy. In the present study, we also found that HIF-1α levels were significantly reduced in the hearts of HFD mice. Surprisingly, knockout of PHD2 did not rescue HFD-induced impairment of HIF-1α expression. The data indicate that sustained activation of PHD2 may not contribute to obesity-related reduction of HIF-1α.

Recent studies suggest an involvement of MYD88 in HFD-induced obesity and inflammation [38], [39]. Knockout of MyD88 has been shown to attenuate HFD-induced weight gain and leptin resistance in mice. Deletion of MyD88 further improves HFD-induced impairment of glucose tolerance [38]. MYD88 also has been shown to be involved in HFD-induced inflammation in the liver [39]. Knockout of MYD88 further attenuates cardiac hypertrophy, inflammation and cell apoptosis via inhibition of NF-κb signaling pathway in post-myocardial infarction [24]. Previous studies also show that both pharmacologic and genetic inhibition of MYD88 attenuate cardiac hypertrophy and apoptosis in myocardial infarction and pressure-overload mouse models in a TLR-NF-κb dependent manner [23], [40], [41]. To our knowledge, this study was the first report to demonstrate that MYD88 was significantly upregulated in the hearts of HFD fed mice. Moreover, knockout of PHD2 inhibited MYD88 expression, reduced myocardial apoptosis and macrophage infiltration. This was accompanied by a significant suppression of TNFα and ICAM-1 expression. Our data further highlight a critical role of PHD2 in HFD-induced activation of MYD88-NF-κb signaling and inflammation.

Toll-like receptors (TLR), a family of pattern-recognition receptors, play an important role in the innate immune system. Activation of inflammatory pathways through TLR4 signaling represents a key step in the development of insulin resistance in obesity [17], [18]. Our present data also showed that inhibition of PHD2 suppressed Toll-like receptor-4 (TLR4) expression and their target genes IRAK4, TNFα and ICAM-1 expression. This was accompanied by a dramatic reduction of macrophage infiltration in the hearts of HFD mice. TLR4 has been reported to induce myocardial hypertrophy via activation of MYD88 and NF-κB pathway [23], [42]. TLR is linked to NF-κB activation and upregulation of pro-inflammatory cytokines such as TNFα [43], [44]. TLR is expressed in many cell types including macrophages, adipocytes and skeletal muscle cells. TLR4 levels were upregulated in the specimen of human heart failure and ischemic hearts [45]–[47]. Knockout of TLR4 has been shown to reduce pressure overload-induced cardiac hypertrophy in mice [42]. Activation of TLR4 in adipose tissue has contributed to obesity-induced inflammation and insulin resistance [48]–[50]. Moreover, knockout of TLR4 in adipocytes reduced free fatty acid-induced inflammatory cytokines (TNFα and IL-6) production [48]. Our previous study showed that mice fed a HFD significantly increased TLR4 expression in the vascular tissue. Inhibition of NADPH oxidase attenuated TLR4 expression together with a significant improvement of vascular function in HFD fed mice [20]. Taken together, our present study suggests that elevated PHD2 is probably a key mediator which activates TLR4/MYD88/NF-κb/IRAK-4/TNFα/ICAM-1 signaling pathway in the obese heart.

In summary, our present study demonstrated that PHD2 was persistently actived in HFD mouse hearts. Inhibition of PHD2 attenuated HFD-induced cardiac dysfunction via suppression of TLR4-MYD88-NFκb pathway and inflammation. Our data suggest that PHD2 is an important mediator of obesity-associated cardiomyopathy and is a promising therapeutic target for obesity and its complications.

## References

[1] Semenza GL (2007) Hypoxia-inducible factor 1 (HIF-1) pathway. Sci STKE 2007:cm8 doi:10.1126/stke.4072007cm8. 17925579

[2] Willam C, Maxwell PH, Nichols L, Lygate C, Tian YM, et al (2006) HIF prolyl hydroxylases in the rat; organ distribution and changes in expression following hypoxia and coronary artery ligation. J Mol Cell Cardiol 41:68–77 doi:10.1016/j.yjmcc.2006. 16765982

[3] Appelhoff RJ, Tian YM, Raval RR, Turley H, Harris AL, et al (2004) Differential function of the prolyl hydroxylases PHD1, PHD2, and PHD3 in the regulation of hypoxia-inducible factor. J Biol Chem 279:38458–38465 doi:10.1074/jbc.M406026200. 15247232

[4] Fong GH, Takeda K (2008) Role and regulation of prolyl hydroxylase domain proteins. Cell Death Differ 15:635–641 doi:10.1038/cdd.2008. 18259202

[5] Nwogu JI, Geenen D, Bean M, Brenner MC, Huang X, et al (2001) Inhibition of collagen synthesis with prolyl 4-hydroxylase inhibitor improves left ventricular function and alters the pattern of left ventricular dilatation after myocardial infarction. Circulation 104:2216–2221 doi:10.1161/hc4301.097193. 11684634

[6] Matsuura H, Ichiki T, Inoue E, Nomura M, Miyazaki R, et al (2013) Prolyl hydroxylase domain protein 2 plays a critical role in diet-induced obesity and glucose intolerance. Circulation 127:2078–2087 doi:10.1161/CIRCULATIONAHA.113.001742. 23630130

[7] Miyata T, de SC (2010) Diabetic nephropathy: a disorder of oxygen metabolism? Nat Rev Nephrol 6:83–95 doi:10.1038/nrneph.2009.211. 20010896

[8] Taniguchi CM, Finger EC, Krieg AJ, Wu C, Diep AN, et al (2013) Cross-talk between hypoxia and insulin signaling through Phd3 regulates hepatic glucose and lipid metabolism and ameliorates diabetes. Nat Med 19:1325–1330 doi:10.1038/nm.3294. 24037093 PMC4089950

[9] Zhang H, Zhang G, Gonzalez FJ, Park SM, Cai D (2011) Hypoxia-inducible factor directs POMC gene to mediate hypothalamic glucose sensing and energy balance regulation. PLoS Biol 9:e1001112 doi:10.1371/journal.pbio.1001112. 21814490 PMC3144184

[10] Cummins EP, Berra E, Comerford KM, Ginouves A, Fitzgerald KT, et al (2006) Prolyl hydroxylase-1 negatively regulates IkappaB kinase-beta, giving insight into hypoxia-induced NFkappaB activity. Proc Natl Acad Sci U S A 103:18154–18159 doi:10.1073/pnas.0602235103. 17114296 PMC1643842

[11] Fujita N, Gogate SS, Chiba K, Toyama Y, Shapiro IM, et al (2012) Prolyl hydroxylase 3 (PHD3) modulates catabolic effects of tumor necrosis factor-alpha (TNF-alpha) on cells of the nucleus pulposus through co-activation of nuclear factor kappaB (NF-kappaB)/p65 signaling. J Biol Chem 287:39942–39953 doi:10.1074/jbc.M112.375964. 22948157 PMC3501017

[12] Kiss J, Mollenhauer M, Walmsley SR, Kirchberg J, Radhakrishnan P, et al (2012) Loss of the oxygen sensor PHD3 enhances the innate immune response to abdominal sepsis. J Immunol 189:1955–1965 doi:10.4049/jimmunol.1103471. 22786772 PMC7611627

[13] Takeda K, Ichiki T, Narabayashi E, Inanaga K, Miyazaki R, et al (2009) Inhibition of prolyl hydroxylase domain-containing protein suppressed lipopolysaccharide-induced TNF-alpha expression. Arterioscler Thromb Vasc Biol 29:2132–2137 doi:10.1161/ATVBAHA.109.196071. 19762779

[14] Lorenzo O, Picatoste B, Ares-Carrasco S, Ramirez E, Egido J, et al (2011) Potential role of nuclear factor kappaB in diabetic cardiomyopathy. Mediators Inflamm 2011:652097 doi:10.1155/2011/652097. 21772665 PMC3136091

[15] Rajesh M, Mukhopadhyay P, Batkai S, Patel V, Saito K, et al (2010) Cannabidiol attenuates cardiac dysfunction, oxidative stress, fibrosis, and inflammatory and cell death signaling pathways in diabetic cardiomyopathy. J Am Coll Cardiol 56:2115–2125 doi:10.1016/j.jacc.2010.07.033. 21144973 PMC3026637

[16] Elmarakby AA, Imig JD (2010) Obesity is the major contributor to vascular dysfunction and inflammation in high-fat diet hypertensive rats. Clin Sci (Lond) 118:291–301 doi:10.1042/CS20090395. 19728860 PMC2842481

[17] Kim SJ, Choi Y, Choi YH, Park T (2012) Obesity activates toll-like receptor-mediated proinflammatory signaling cascades in the adipose tissue of mice. J Nutr Biochem 23:113–122 doi:10.1016/j.jnutbio.2010.10.012. 21414767

[18] Schaeffler A, Gross P, Buettner R, Bollheimer C, Buechler C, et al (2009) Fatty acid-induced induction of Toll-like receptor-4/nuclear factor-kappaB pathway in adipocytes links nutritional signalling with innate immunity. Immunology 126:233–245 doi:10.1111/j.1365-2567.2008.02892.x. 18624726 PMC2632685

[19] Takeda K, Aguila HL, Parikh NS, Li X, Lamothe K, et al (2008) Regulation of adult erythropoiesis by prolyl hydroxylase domain proteins. Blood 111:3229–3235 doi:10.1182/blood-2007-09-114561. 18056838 PMC2265459

[20] Chen JX, Stinnett A (2008) Critical role of the NADPH oxidase subunit p47(phox) on vascular TLR expression and neointimal lesion formation in high-fat diet-induced obesity. Lab Invest. 88(12):1316–28 doi:10.1038/labinvest.2008.92. 18779779

[21] Rottman JN, Ni G, Brown M (2007) Echocardiographic evaluation of ventricular function in mice. Echocardiography 24:83–89 doi:10.1111/j.1540-8175.2006.00356.x. 17214630

[22] Zeng H, Li L, Chen JX (2012) Overexpression of Angiopoietin-1 Increases CD133+/c-kit+ Cells and Reduces Myocardial Apoptosis in db/db Mouse Infarcted Hearts. PLoS One 7:e35905 doi:10.1371/journal.pone.0035905. 22558265 PMC3338852

[23] Li T, Wang Y, Liu C, Hu Y, Wu M, et al (2009) MyD88-dependent nuclear factor-kappaB activation is involved in fibrinogen-induced hypertrophic response of cardiomyocytes. J Hypertens 27:1084–1093 doi:10.1097/HJH.0b013e3283293c93. 19342961

[24] Singh MV, Swaminathan PD, Luczak ED, Kutschke W, Weiss RM, et al (2012) MyD88 mediated inflammatory signaling leads to CaMKII oxidation, cardiac hypertrophy and death after myocardial infarction. J Mol Cell Cardiol 52:1135–1144 doi:10.1016/j.yjmcc.2012.01.021. 22326848 PMC3327770

[25] Takeda K, Cowan A, Fong GH (2007) Essential role for prolyl hydroxylase domain protein 2 in oxygen homeostasis of the adult vascular system. Circulation 116:774–781 doi:10.1161/CIRCULATIONAHA.107.701516. 17646578

[26] Chen JX, Stinnett A (2008) Ang-1 gene therapy inhibits hypoxia-inducible factor-1alpha (HIF-1alpha)-prolyl-4-hydroxylase-2, stabilizes HIF-1alpha expression, and normalizes immature vasculature in db/db mice. Diabetes 57:3335–3343 doi:10.2337/db08-0503. 18835934 PMC2584141

[27] Lin Q, Huang Y, Booth CJ, Haase VH, Johnson RS, et al (2013) Activation of hypoxia-inducible factor-2 in adipocytes results in pathological cardiac hypertrophy. J Am Heart Assoc 2:e000548 doi:10.1161/JAHA.113.000548. 24326162 PMC3886757

[28] Van der Heiden K, Cuhlmann S, Luong lA, Zakkar M, Evans PC (2010) Role of nuclear factor kappaB in cardiovascular health and disease. Clin Sci (Lond) 118:593–605 doi:10.1042/CS20090557. 20175746

[29] Valen G, Yan ZQ, Hansson GK (2001) Nuclear factor kappa-B and the heart. J Am Coll Cardiol 38:307–314 doi:10.1016/S0735-1097(01)01377-8. 11499717

[30] Ritchie ME (1998) Nuclear factor-kappaB is selectively and markedly activated in humans with unstable angina pectoris. Circulation 98:1707–1713 doi:10.1161/01.CIR.98.17.1707. 9788823

[31] Maier HJ, Schips TG, Wietelmann A, Kruger M, Brunner C, et al (2012) Cardiomyocyte-specific IkappaB kinase (IKK)/NF-kappaB activation induces reversible inflammatory cardiomyopathy and heart failure. Proc Natl Acad Sci U S A 109:11794–11799 doi:10.1073/pnas.1116584109. 22753500 PMC3406816

[32] Hamid T, Guo SZ, Kingery JR, Xiang X, Dawn B, et al (2011) Cardiomyocyte NF-kappaB p65 promotes adverse remodelling, apoptosis, and endoplasmic reticulum stress in heart failure. Cardiovasc Res 89:129–138 doi:10.1093/cvr/cvq274. 20797985 PMC3002872

[33] Alvarez-Guardia D, Palomer X, Coll T, Davidson MM, Chan TO, et al (2010) The p65 subunit of NF-kappaB binds to PGC-1alpha, linking inflammation and metabolic disturbances in cardiac cells. Cardiovasc Res 87:449–458 doi:10.1093/cvr/cvq080. 20211864

[34] Frantz S, Hu K, Bayer B, Gerondakis S, Strotmann J, et al (2006) Absence of NF-kappaB subunit p50 improves heart failure after myocardial infarction. FASEB J 20:1918–1920 doi:10.1096/fj.05-5133fje. 16837548

[35] Kawamura N, Kubota T, Kawano S, Monden Y, Feldman AM, et al (2005) Blockade of NF-kappaB improves cardiac function and survival without affecting inflammation in TNF-alpha-induced cardiomyopathy. Cardiovasc Res 66: 520–529. 10.1016/j.cardiores.2005.02.007 15914117

[36] Valen G (2004) Signal transduction through nuclear factor kappa B in ischemia-reperfusion and heart failure. Basic Res Cardiol 99:1–7 doi:10.1007/s00395-003-0442-7. 14685699

[37] Mou SS, Haudek SB, Lequier L, Pena O, Leonard S, et al (2002) Myocardial inflammatory activation in children with congenital heart disease. Crit Care Med 30:827–832. 11940753 10.1097/00003246-200204000-00018

[38] Kleinridders A, Schenten D, Konner AC, Belgardt BF, Mauer J, et al (2009) MyD88 signaling in the CNS is required for development of fatty acid-induced leptin resistance and diet-induced obesity. Cell Metab 10:249–259 doi:10.1016/j.cmet.2009.08.013. 19808018 PMC3898351

[39] Yokoyama S, Hosoi T, Ozawa K (2012) Stearoyl-CoA Desaturase 1 (SCD1) is a key factor mediating diabetes in MyD88-deficient mice. Gene 497:340–343 doi:10.1016/j.gene.2012.01.024. 22326531

[40] Ha T, Hua F, Li Y, Ma J, Gao X, et al (2006) Blockade of MyD88 attenuates cardiac hypertrophy and decreases cardiac myocyte apoptosis in pressure overload-induced cardiac hypertrophy in vivo. Am J Physiol Heart Circ Physiol 290: H985–H994. 10.1152/ajpheart.00720.2005 16199478

[41] Van Tassell BW, Seropian IM, Toldo S, Salloum FN, Smithson L, et al (2010) Pharmacologic inhibition of myeloid differentiation factor 88 (MyD88) prevents left ventricular dilation and hypertrophy after experimental acute myocardial infarction in the mouse. J Cardiovasc Pharmacol 55:385–390 doi:10.1097/FJC.0b013e3181d3da24. 20125030

[42] Ha T, Li Y, Hua F, Ma J, Gao X, et al (2005) Reduced cardiac hypertrophy in toll-like receptor 4-deficient mice following pressure overload. Cardiovasc Res 68:224–234 doi:10.1016/j.cardiores.2005.05.025. 15967420

[43] Andreakos E, Sacre S, Foxwell BM, Feldmann M (2005) The toll-like receptor-nuclear factor kappaB pathway in rheumatoid arthritis. Front Biosci 10:2478–2488. 15970510 10.2741/1712

[44] Brown MA, Jones WK (2004) NF-kappaB action in sepsis: the innate immune system and the heart. Front Biosci 9:1201–1217. 14977537 10.2741/1304

[45] Birks EJ, Felkin LE, Banner NR, Khaghani A, Barton PJ, et al (2004) Increased toll-like receptor 4 in the myocardium of patients requiring left ventricular assist devices. J Heart Lung Transplant 23:228–235 doi:10.1016/S1053-2498(03)00106-2. 14761771

[46] Satoh M, Shimoda Y, Maesawa C, Akatsu T, Ishikawa Y, et al (2006) Activated toll-like receptor 4 in monocytes is associated with heart failure after acute myocardial infarction. Int J Cardiol 109:226–234 doi:10.1016/j.ijcard.2005.06.023. 16051384

[47] Frantz S, Ertl G, Bauersachs J (2008) Toll-like receptor signaling in the ischemic heart. Front Biosci 13:5772–5779. 18508620 10.2741/3114

[48] Shi H, Kokoeva MV, Inouye K, Tzameli I, Yin H, et al (2006) TLR4 links innate immunity and fatty acid-induced insulin resistance. J Clin Invest 116:3015–3025 doi:10.1172/JCI28898. 17053832 PMC1616196

[49] Tsukumo DM, Carvalho-Filho MA, Carvalheira JB, Prada PO, Hirabara SM, et al (2007) Loss-of-function mutation in TLR4 prevents diet-induced obesity and insulin resistance. Diabetes 56(8):1986–98 doi:10.2337/db06-1595. 17519423

[50] Kim F, Pham M, Luttrell I, Bannerman DD, Tupper J, et al (2007) Toll-like receptor-4 mediates vascular inflammation and insulin resistance in diet-induced obesity. Circ Res 100:1589–1596 doi:10.1161/CIRCRESAHA.106.142851. 17478729

